# Endocrine and metabolic complications in children and adolescents with Sickle Cell Disease: an Italian cohort study

**DOI:** 10.1186/s12887-019-1423-9

**Published:** 2019-02-11

**Authors:** V. Mandese, E. Bigi, P. Bruzzi, G. Palazzi, B. Predieri, L. Lucaccioni, M. Cellini, L. Iughetti

**Affiliations:** 1 Post Graduate School of Pediatrics, Department of Medical and Surgical Sciences for Mothers, Children and Adults, University of Modena and Reggio Emilia, Via del Pozzo 71, 41124 Modena, Italy; 2 Oncology and Hematology Pediatric Unit Department of Medical and Surgical Sciences for Mothers, Children and Adults, University of Modena and Reggio Emilia, 41124 Modena, Italy; 3 Pediatric Unit, Department of Medical and Surgical Sciences for Mothers, Children and Adults, University of Modena and Reggio Emilia, 41124 Modena, Italy

**Keywords:** Sickle cell disease, Metabolism, Endocrine complications, Children and adolescents

## Abstract

**Background:**

Children with Sickle Cell Disease (SCD) show endocrine complications and metabolic alterations. The physiopathology of these conditions is not completely understood: iron overload due to chronic transfusions, ischemic damage, and inflammatory state related to vaso-occlusive crises may be involved. Aims of this study were to evaluate the growth pattern, endocrine complications, and metabolic alterations and to detect the relationship between these conditions and the SCD severity in affected children and adolescents.

**Methods:**

Fifty-two children and adolescents with SCD [38 homozygous sickle hemoglobin (HbSS) and 14 heterozygous sickle hemoglobin (HbSC); age range 3–18 years] were recruited. Anthropometric [height, body mass index (BMI), arm span, sitting height, target height (TH), and pubertal status] and laboratory [blood cell counts, hemolysis indices, metabolic and nutritional status indices and hormonal blood levels] data were evaluated. The SCD severity was defined according to hematological and clinical parameters.

**Results:**

Height-SDS adjusted for TH and BMI-SDS were significantly higher in HbSC children than in HbSS ones. Forty-eight out of 52 patients (92%) had at least one metabolic and/or endocrine alteration: insufficiency/deficiency of vitamin D (84.7%), insulin resistance (11.5%), growth hormone deficiency (3.8%), subclinical hypothyroidism (3.8%), and hypogonadism (1.9%). Levels of vitamin D were significantly and negatively correlated with clinical indicators of the SCD severity. Subjects with HbSS genotype show significant lower levels of both insulin-like growth factor-1 (IGF-1) and insulin-like growth factor binding protein 3 than children with HbSC. In the study population IGF-1 values were significantly and positively correlated with Hb and negatively with lactate dehydrogenase.

**Conclusions:**

Metabolic alterations and endocrine complications are very common in children and adolescents with SCD. A regular follow-up is necessary to identify subjects at risk for complications to precociously start an appropriate treatment and to improve the quality of life of SCD patients.

**Electronic supplementary material:**

The online version of this article (10.1186/s12887-019-1423-9) contains supplementary material, which is available to authorized users.

## Background

Sickle cell disease (SCD) is an inherited disease due to a single-point mutation on the β-globin subunit of hemoglobin (Hb) determining polymerization of the mutant HbS and resulting in sickling of erythrocytes. It is characterized by a high clinical variability because of inflammation, hemolysis, and micro-vascular obstruction leading to unpredictable acute complications and chronic organ damage [1, 2]. The HbS mutation can be inherited in homozygosis (HbSS) or in heterozygosis with other β-globin qualitative (HbSC) or quantitative (HbSβ0 and HbSβ+) defects. Subjects affected by HbSS and HbSβ0 have the most severe phenotype while the other forms have milder clinical manifestations [3].

In high-income countries the great and continuous rise of the SCD survival rate demonstrated in the last decades was mainly due to newborn screening programs [4–6], advances in the supportive care, and a better use of disease modifying agents such as Hydrxyurea (HU) [7]. However, the reduction of mortality has led to an increase of long-term complications, including also metabolic and endocrine ones.

Specifically, poor growth and delay of pubertal development are the most frequent disorders observed in children and adolescent with SCD. Children with SCD have lower height, weight, and body mass index (BMI) than healthy controls [8]. However, published data on endocrine and metabolic disorders during childhood and adolescence, such as gonadal insufficiency, thyroid dysfunction, and bone and glycemic metabolism, are really few [9–11]. The pathophysiology of these complications is not yet fully understood. Endocrine disorders appear to be related to vaso-occlusive and ischemic events, rather than iron overload resulting from frequent transfusion [9]. According to the literature, the prevalence of endocrine and metabolic disorders in children with SCD varies in different populations depending on the literacy rate, socioeconomic status, and access to appropriate treatment [7, 9, 10, 12]. In children with HbSS, the treatment with HU has been demonstrated to allow growth rates similar to patients with HbSβ^+^ or healthy controls [13].

Aims of the present study were to define the growth pattern, endocrine complications, and metabolic alterations in children and adolescents with SCD and to evaluate the role of therapeutic regimens in improving anthropometric, endocrine, and metabolic parameters.

## Methods

### Study design and setting

This was a cross-sectional population study. We evaluated 52 children and adolescents with SCD (38 with HbSS and 14 with HbSC) at steady state, aged between 3 and 18 years, who were recruited during the first six months of 2017.

Patients with acute complications or comorbidities (genetic disease, congenital heart disease, neurological disease), lost to follow-up or transferred to other centers were excluded.

The study was approved by the Ethics Committee of the University of Modena and Reggio Emilia (Protocol number 213/16). Written informed consent was obtained from all parents at the moment of recruitment in the study and before the first data collection. The study database was created before the beginning of patient’s recruitment and was approved by the local EC before data collection.

### Data collection

Anthropometric parameters [height, weight, body mass index (BMI), arm span, sitting height] were evaluated in all recruited subjects. Height and sitting height were measured to the nearest 0.1-cm with a wall-mounted stadiometer and stadiometer for sitting height (Harpenden, Crymych; UK), respectively. Body weight was measured to the nearest 0.1-kg. Arm span was represented by the distance, measured in cm, between the end of the third finger of the two hands and it was measured to the nearest 0.1-cm with a no extensible meter. We calculated: sitting height/height ratio, arm span/height ratio, and BMI (weight in kg/height in meters squared).

Height-SDS and BMI-SDS were reported according to age- and sex- specific World Health Organization (WHO) growth chart 2007 [14]. Parental height was also collected to estimate target height (TH) according to the formula: [(mother’s height + 13) + father’s height]/2 in males and [(mother’s height - 13) + father’s height]/2 in females [15]. In all the participants pubertal development was determined using the grading system defined by Tanner for breast (B) and genital stage (G) according to gender [16].

Blood and plasma samples were collected in all SCD children to measure: blood cell counts [red blood cells, white blood cells (WBC), neutrophils (N), hemoglobin (Hb), platelets (PTL)], lactate dehydrogenase (LDH) as hemolysis index, iron levels, metabolic and nutritional status indices [fasting glucose (enzymatic test Gluco-Quant, Roche), fasting insulin (chemiluminescent immunometric assay, Immunolite 2000, Siemens healthcare), lipid status [total cholesterol, high-density lipoprotein (HDL-C), low-density lipoprotein, tryglicerides) (enzymatic test Hitachi, Roche Diagnostic)], thyroid hormones [thyroid-stimulating hormone (TSH) and free thyroxine 4 (fT4) (fluorometric assay AutoDELFIA automatic immune assay system)], vitamin D (chemiluminescent immunometric assay, BAYER, Germany), reproductive and growth function [luteinizing hormone (LH), follicle stimulating hormone (FSH), prolactin, estradiol, testosterone, insulin-like growth factor-1 (IGF-1), (chemiluminescent immunometric assay, BAYER, Germany), and insulin-like growth factor binding protein-3 (IGFBP-3) (ELISA test)].

Vitamin D insufficiency and deficiency were defined by 25-hydroxy-vitamin D levels between 10 and 30 ng/ml and < 10 ng/ml, respectively.

Insulin resistance was estimated using the homeostasis model assessment (HOMA) model as fasting insulin (microU/L) x fasting glucose (mmol/L)/22.5. Insulin resistance was defined by HOMA-IR values of ≥3.16 in pubertal subjects and HOMA-IR of ≥2.67 in prepubertal ones [17, 18].

Subclinical hypothyroidism was defined by normal FT4 values associated to TSH > 5 μIU/ml [19].

Growth hormone (GH) deficiency (GHD) was diagnosed according to both specific anthropometric (height < − 3 SDS or height < − 2 SDS associated with height growth velocity < -1SDS) and biochemical parameters (GH peak values < 10 ng/ml in 2 different pharmacologic stimulation tests) [20].

Hypergonadotropinic hypogonadism was defined by high serum gonadotropin concentrations in the absence of pubertal signs at the appropriate age for puberty [21].

Ovarian insufficiency was defined in post pubertal female with secondary amenorrhea, high concentration of FSH and low anti-mullerian hormone (AMH) levels [21].

The severity of SCD was evaluated according to the following indices: the average of total Hb and LDH (year 2016), the average number of hospitalizations and days of hospitalization (year 2016), the average number of hospitalizations of the last five years, and the total number of lifetime Acute Chest Syndrome (ACS) episodes.

### Statistical analysis

Descriptive data are reported as mean ± standard deviation (SD), number of observations, and percentages. Data were checked for normal distribution using the Kolmogorov-Smirnov test, so non-parametric statistical analysis (STATISTICA™ software, StatSoft Inc., Tulsa, OK, USA) was performed.

Subjects’ data were analyzed according to gender (males vs. females), SCD genotype (HbSC vs. HbSS), HDL-C levels (cut-off 40 mg/dl), and HU treatment (HU > 1 year vs. HU < 1 year).

Between-group comparisons were performed using the Mann-Whitney *U*-test. Spearman correlation was used to evaluate correlations between clinical, anthropometric, and biochemical parameters.

For each test, statistical significance was considered for *p* < 0.05.

## Results

This study reports data from 52 children and adolescents (29 males, 55.7%) with SCD having mean age of 11.1 ± 4.6 years. Thirty-eight subjects (73%) presented HbSS genotype while others were HbSC. Among our population, 50% of patients was pubertal; 9 out of 23 female had menarche (average age of menarche 12.8 years). Analyzing patients according to the genotype (HbSS and HbSC), 42% of subjects with HbSS and 70% of subjects with HbSC were pubertal.

HbSS showed lower level of Hb and higher level of HbS %, WBC, PTL, LDH, and bilirubin than HbSC ones (Table 1).

**Table 1:** Laboratory data in HbSS patients vs. HbSC patients

Laboratory data	Hb SS	HbSC	*P*-value
	Group (*n* = 38)	Group (*n* = 14)	
HU > 1 years (%)	71% (27/38)	21.4% (3/14)	–
WBCs, 1000s	11.2 ± 4.31	6.76 ± 1.80	**0.0001**
WBCs, 1000s(mean 2016)	11.2 ± 3.32	7.18 ± 1.96	**< 0.0001**
Neutrophils, %	50.0 ± 12.1	49.9 ± 10.6	0.9835
Neutrophils, % (mean 2016)	49.1 ± 9.9	47.7 ± 8.3	0.4960
Hb, g/dl	9.0 ± 1.0	11.8 ± 1.2	**< 0.0001**
Hb, g/dl (mean 2016)	9.1 ± 0.9	11.6 ± 1.2	**< 0.0001**
Hb S, %	63.3 ± 14.2	46.7 ± 10.4	**0.0005**
Hb F, %	15.7 ± 7.8	7.0 ± 11.5	**0.0003**
Platelets, 1000s	421 ± 201	221 ± 100	**0.0002**
LDH, U/L	951.8 ± 216.5	582.4 ± 144.8	**< 0.0001**

Data are reported as mean ± SD (standard deviation)

*Abbreviations*: *HbSS* homozygous SS patients, *HbSC* double heterozygous SC patients, *HU* hydroxyurea, *WBC* white blood cells, *Hb* hemoglobin, *LDH* lactate dehydrogenase

*P*-values statistically significant are printed in bold

### Anthropometric parameters

**Fig. 1:**
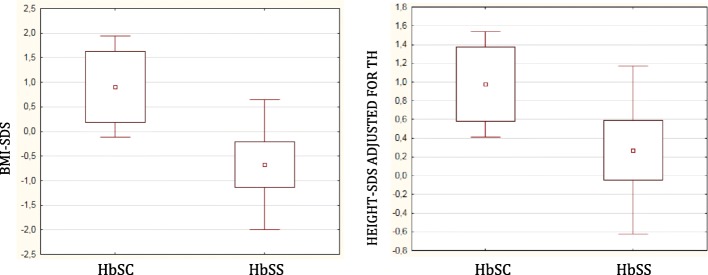
Anthropometric parameters according to SCD genotype. BMI-SDS in HbSC group was significantly higher than in HbSS group (*p* = 0.004). Height-SDS adjusted for TH in HbSC group was significantly higher than in HbSS group (*p* = 0.027)

Subjects with HbSC genotype compared to HbSS ones showed significantly higher values of both height-SDS adjusted for TH (1.0 ± 0.6 vs. 0.3 ± 0.9 SDS, respectively; *p* = 0.027) and BMI-SDS (0.9 ± 1.1 vs. -0.7 ± 1.4 SDS, respectively; *p* = 0.004) despite chronological age was not different (Table 2; Fig. 1). Analyzing data according to gender no difference was found in anthropometric parameters (Additional file 1: Table S1).

**Table 2:** Anthropometric parameters in HbSS patients vs. HbSC patients

Anthropometric data	Hb SS	HbSC	P-value
	Group (*n* = 38)	Group (*n* = 14)	
Age (years)	10.44 ± 4.55	13.05 ± 4.47	0.0850
Height	137.1 ± 21.7	150.2 ± 24.5	0.1340
Height-SDS	− 0.2 ± 1.1	0.4 ± 0.7	0.1807
Height-SDS adjusted for TH	0.3 ± 0.9	1.0 ± 0.6	**0.0270**
Weight	32.6 ± 14.0	51.1 ± 25.0	**0.0374**
BMI (Kg/m^2^)	16.5 ± 2.7	21.1 ± 4.9	**0.0045**
BMI-SDS	−0.7 ± 1.4	0.9 ± 1.1	**0.0043**
Growth velocity cm/year	4.0 ± 2.3	4.2 ± 3.3	0.7863
Growth velocity -SDS	−1.6 ± 2.2	−2.3 ± 3.5	0.8398
Sitting height	69.3 ± 9.0	75.7 ± 11.8	0.1760
Sitting height/height	0.51 ± 0.02	0.50 ± 0.02	0.8614

Data are reported as mean ± SD (standard deviation)

*Abbreviations*: *HbSS* homozygous SS patients, *HbSC* double heterozygous SC patients, *SDS* standard deviation, *TH* target height, *BMI* body mass index

*P*-values statistically significant are printed in bold

Height-SDS adjusted for TH was significantly and negatively correlated with clinical severity parameters such as number of hospital admissions/2016 (Spearman R = − 0.31 *p* = 0.040), average number of days of hospital admissions/2016 (Spearman R = − 0.31, *p* = 0.041), and average number of ACS (Spearman R = − 0.40, *p* = 0.008).

**Fig. 2:**
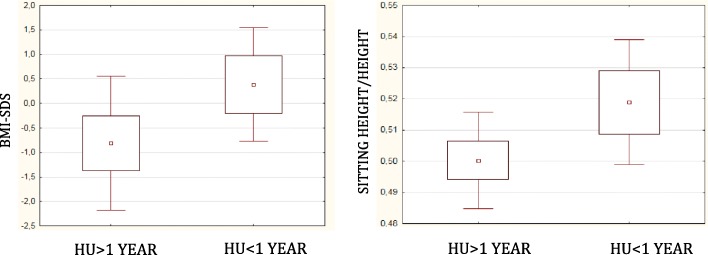
Anthropometric parameters according to HU therapy groups. Patients in HU > 1-year group, respect to HU < 1-year one, had both significantly lower BMI-SDS (*p* = 0.008) and sitting height/height ratio (*p* = 0.004)

Two out of 52 of SCD subjects (3.8%) showed height-SDS < − 2 SDS and 9.6% (5/52) showed BMI-SDS < − 2 SDS. Patients on treatment with HU for more than one year (29/52, 56%) respect to those on HU for less time had lower values of BMI-SDS ​​ (− 0.8 ± 1.4 vs. 0.4 ± 1.2 SDS, respectively; *p* = 0.008) and sitting height/height ratio (0.50 ± 0.02 vs. 0.52 ± 0.02, respectively; *p* = 0.004) (Fig. 2).

### Prevalence of metabolic and endocrine complications

The prevalence of metabolic alterations and endocrine complications among SCDs was high: 48 out of 52 patients show at least one metabolic and/or endocrine alteration. Among all patients, 41 (79%), 6 (11.5%), and 1 (1.9%) presented respectively one, two, and three alterations at the same time. The most detected conditions were the vitamin D insufficiency/deficiency (84.7%), the insulin resistance (11.5%), and to a lesser extent the GHD (3.8%), the subclinical hypothyroidism (3.8%), and the hypergonadotropic hypogonadism (1.9%) (Table 3).

**Table 3:** Prevalence of endocrine and metabolic alterations in children and adolescents with SCD

Endocrine/metabolic complications	N°/52	%	M/F	SS/SC
Vitamin D insufficiency (10–30 ng/ml)	33	63.5%	16/17	24/9
Vitamin D deficiency (< 10 ng/ml)	11	21.2%	7/4	7/4
GHD	2	3.8%	2/0	2/0
Subclinical hypothyroidism	2	3.8%	1/1	2/0
Hypergonadotropinic hypogonadism	1	1.9%	1/0	1/0
Ovarian insufficiency	1	1.9%	0/1	1/0
Insulin resistance	6	11.5%	2/4	4/2

*Abbreviations*: *GHD* growth deficiency hormone

### Vitamin D insufficiency/deficiency

In particular, in 63.5% of patients vitamin D levels were between 10 and 30 ng/ml while in 21.2% were < 10 ng/ml. We found a significant negative relationship between plasmatic levels of vitamin D and clinical severity of the disease, represented by number of hospital admissions/2016 (Spearman R = − 0.29 *p* = 0.040), average number of days of hospital admissions/2016 (Spearman R = − 0.29, *p* = 0.034) and average number of hospital admissions in the last 5 years (Spearman R = − 0.36, *p* = 0.009).

### Glucose and lipid metabolism

**Fig. 3:**
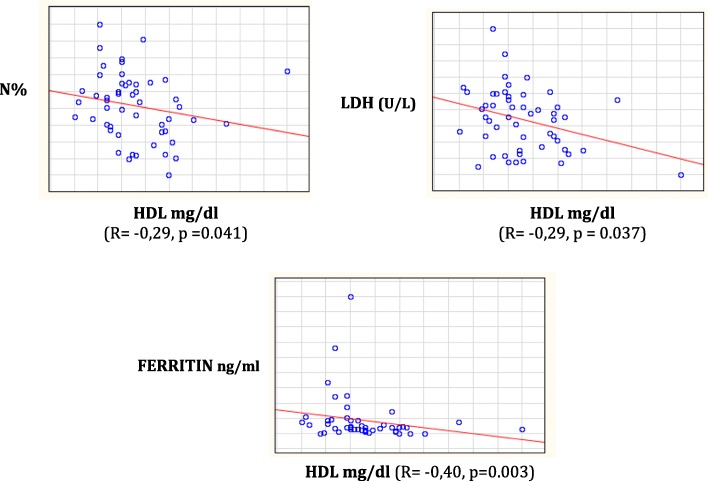
Relationship between HDL-C values and parameters of clinical severity

Analyzing data according to HDL-C levels, we found that subjects with HDL-C > 40 mg/dl, respect to those with HDL-C < 40 mg/dl, had significantly higher levels of vitamin D (22.4 ± 11.2 vs. 18.2 ± 17.3 ng/ml, respectively; *p* = 0.044). The mean values of HDL-C were correlated with neutrophils (Spearman R = − 0.29, *p* = 0.041), LDH (Spearman R = − 0.29, *p* = 0.037), and serum ferritin (Spearman R = − 0.40, *p* = 0.003) (Fig. 3).

The 11.5% of subjects had insulin resistance as suggested by abnormal HOMA-IR values. However, HOMA-IR was not different between HbSS and HbSC subjects.

### Growth and gonadotropin

GHD was detected in 2 boys (3.8%) with HbSS genotype, who have been started human recombinant GH replacement therapy.

**Fig. 4:**
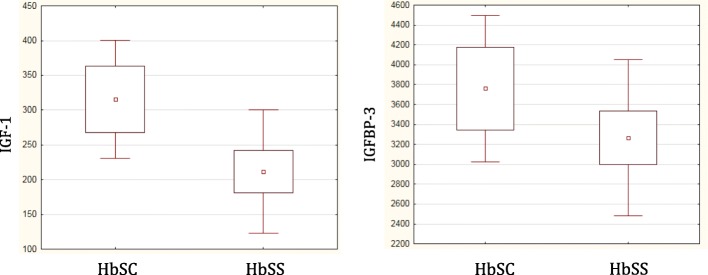
IGF-1 and IGFBP-3 values according to SCD genotype. In HbSC group both IGF-1 and IGFBP-3 levels were significantly higher respect to HbSS group (*p* < 0.0001)

**Fig. 5:**
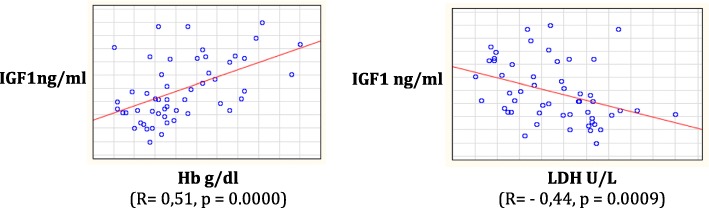
Relationship between values of IGF-1 and parameters of clinical severity

In addition, patients with HbSS genotype compared to HbSC ones showed lower levels of IGF-1 (211.7 ± 93.2 vs. 315.3 ± 89.3 ng/ml, respectively; *p* < 0.001) and IGFBP-3 (3267.1 ± 828, 4 vs. 3761.7 ± 773.5 ng/ml, respectively; *p* < 0.001) (Fig. 4). IGF-1 values were significantly correlated with both Hb (Spearman R = 0.51, *p* = 0.0001) and LDH levels (Spearman R = − 0.44, *p* = 0.0009) (Fig. 5).

Ovarian insufficiency was detected in one 17-years old girl with normal secondary sexual characteristics for age, with secondary amenorrhea, high concentration of FSH and low levels of AMH.

Diagnosis of hypergonadotropic hypogonadism was also performed in a 15-years old male with HbSS genotype and with Tanner Stage 1 (testes 3 ml bilateral).

The mean values ​​of testosterone in our males were also positively correlated with the mean values ​​of Hb (Spearman R = 0.40, *p* = 0.029). No correlation between IGF-1, IGFBP-3, TSH, fT4, testosterone, estradiol, LH, FSH levels and ferritin values [both as a relative value at the time of enrollment and as the average value of the last two years (2015–2017)] were identified in our population both in prepubertal and in pubertal patients.

## Discussion

Survival rate among children with SCD has increased especially in the recent decades, due to an earlier diagnosis and a better quality of care. Consequently, the incidence of long-term complications, such as metabolic and endocrine disorders, is increasing in SCD population and it has become a main concern to treat them properly, improving their prognosis and their quality of life. In this study we demonstrated a high prevalence (92%) of endocrine complications and metabolic alterations in the pediatric SCD population, mainly represented by vitamin D insufficiency or deficiency, insulin resistance, and to a lesser extent GHD, subclinical hypothyroidism, and hypogonadism. In literature, it is really difficult to understand the cumulative incidence of these disorders in subjects with SCD because of published studies evaluated mainly one single endocrine alteration. Specifically, growth impairment and delayed puberty are the most frequent disorders observed among SCD pediatric patients [10, 11].

*Özen* et al. reported that 50% of the examined population show endocrine disorders mainly represented, as in our study, by insufficiency/deficiency of vitamin D and to a lesser degree of osteopenia, hypoplasia/testicular atrophy, hypogonadism, hypothyroidism, and insulin resistance [22].

In our study the prevalence of endocrine complications was even higher that those reported by *Özen* et al. [22], but it is important to consider that the majority of our subjects were immigrants, coming mainly from Africa (96%) with socio-economic conditions that may influence the anthropometric, endocrine and metabolic parameters.

It has been demonstrated that children with SCD had a poorer growth compared to matched healthy subjects [23]. Near two thirds of SCD patients experience a decline in one or more growth parameters (height, weight, and BMI) and the incidence of growth retardation (defined by the presence of one or more of anthropometric parameters below the 5th percentile) could reach the 38% during the follow-up [24]. In our population, the prevalence of growth alterations was about 3.8% when height was considered <-2DS ​​and 9.6% when BMI-SDS was considered <-2SD. The discrepancy between our results and previous published data [24] could be explained by differences in the study design (longitudinal vs. transversal study).

The underline mechanism on growth delay in SCD is very complex and probably influenced by many variables, such as hematologic and cardiovascular status, socio-economic factors, endocrine function, metabolic function and nutritional status [25].

It has been shown that the mean height SDS of children with SCD is comparable to those of children with constitutional growth delay but it is higher than those of children with GHD [26, 27]. In agreement with published data, our study demonstrated that growth was more affected in subjects with HbSS genotype than in subjects with HbSC genotype.

According to the therapeutic regimen, significant differences were found with respect to BMI-SDS and sitting height/height ratio. Although not expected, patients treated with HU for more than one year had lower BMI-SDS and sitting height/height ratio. The reason of these findings is likely related to the more severe phenotype of patients treated with HU for more than a year. In fact, in our opinion, the disease severity could influence these anthropometric parameters. However, it would be important to continue these evaluations in order to assess whether treated patients may have an improvement in growth parameters over time by a reduction in clinical severity. In fact, the prospective use of HU can both improve clinical outcome of the disease and also positively influence growth and development, reducing the risk of iron overload due to the chronic transfusion regimen.

Our data showed significant correlations between clinical parameters of disease severity and anthropometric parameters: children with better control of the disease (expressed as lower number of hospital admissions in 2016, lower number of days of hospitalization in 2016 and lower ACS) had higher values of height-SDS adjusted for TH. Subjects with HbSS genotype showed a negative correlation between the number of ACS and the values of height-SDS adjusted for TH. A good clinical control of the disease seems not only to affect the survival but also to reduce the long-term comorbidity.

In our SCD population, vitamin D insufficiency was demonstrated in 63.5% while 21.2% had a deficient level. In a study conducted by *Buison* et al., 65% of children with SCD had levels of vitamin D lower than those of healthy children [28]. *Jackson* et al [29] reported that 96% of SCD patients had vitamin D level between 10 and 20 ng/ml. Severe vitamin D deficiency (< 10 ng/ml) was found in 64% of subjects and it was demonstrated to be associated with age and reduction in lung function but not with pain and/or ACS episodes. In a Spanish study on vitamin D status in 78 children with SCD, *Garrido* et al. [30] report that near to 80% and 56.4% had vitamin D level < 30 ng/ml and < 20 ng/ml, respectively.

The vitamin D metabolism is complex because of the involvement of different organ including skin, intestines, liver, kidney, and parathyroid [9]. Patients with SCD have some peculiar characteristics that can lead to the development of vitamin D deficiency such as decreased appetite or reduction of nutrients absorption due to the intestinal mucosa damage. Continuous red blood cells production to compensate anemia characterize SCD and causes an increase of basal metabolic rate with higher nutritional demands [8, 31]. Moreover, in SCD patients with renal impairment conversion of vitamin D to its active form can be reduced. Finally, vitamin D binding protein levels can be low being SCD an inflammatory disease [28]. The importance of vitamin D assessment in patients with SCD is supported by the demonstration that vitamin D deficiency is more frequent among children with SCD than in controls [32].

The different prevalence of vitamin D deficiency/insufficiency demonstrated between African Americans and Caucasians populations can be explained by the decreased synthesis of vitamin D in the skin [33] and differences in dietary habits [34]. A better absorption of dietary calcium and lower levels of vitamin D binding protein have been demonstrated in African Americans subjects compared to Caucasians [35, 36]. This suggests that neither the optimal Vitamin D threshold for Caucasians nor levels suggested for healthy African Americans are applicable to patients with SCD [32]. It is therefore really important to identify the optimal level of vitamin D in children and adults with SCD, in particular in patients of African origin living in European Countries, as patients enrolled in our study.

In our population, vitamin D levels showed an inverse and statistically significant correlation with the number of admissions and hospitalizations in 2016 and the average number of admissions in the last 5 years, suggesting that this deficit could adversely affects the clinical severity of the disease.

It was hypothesized that inadequate levels of vitamin D could be linked to a condition of chronic inflammation, as well as low levels of HDL-C [37]. This finding is confirmed also in our population study. Dividing our patients into two groups according to HDL-C levels, we found that vitamin D values ​​were significantly lower in the group of patients with lower HDL-C values (< 40 mg/dl). In addition, HDL-C values ​​of our population showed a negative relationship (*p* < 0.05) to neutrophils percentage, LDH and ferritin values, particularly in subjects with HbSS genotype. *Seixas* et al. [37] found a negative association between LDH and HDL-C levels, showing how low HDL-C levels could be a prognostic marker of hemolysis and endothelial dysfunction in view of their anti-inflammatory, anti-oxidant, anti-aggregating, anti-coagulant and pro-fibrinolytic role. Patients with SCD and high HDL-C levels had fewer reticulocytes, WBC, monocytes, PTL, and erythroblasts and a lower concentration of HbS as well as a lower concentration of hemolytic markers. Our data confirm that HDL-C and vitamin D could play an important role in inflammatory condition such as SCD.

The 3.8% of our population showed GHD. An impairment of the GH-IGF1-IGFBP3 axis was demonstrated in SCD subjects [38, 39]. Children with SCD have significantly decreased IGF-1 concentrations compared to children with constitutional delay of growth. The poor synthesis of IGF-1 could depend on a primitive defect of the axis, but also from malnutrition and hypermetabolic status of these patients [40]. In some cases, however, there is a real GHD due to a pituitary vascular insult during vaso-occlusive crises [41, 42]. These patients could benefit from a human recombinant GH replacement therapy [43]. Our data showed that mean values ​​of both IGF-1 and IGFBP-3 were lower in subjects with HbSS genotype compared to subjects with HbSC genotype and that IGF1-levels had a positive correlation with Hb and Hb mean values ​​of 2016 and a negative correlation with average LDH and LDH mean values ​​of 2016. These data underline how the clinical severity of the disease, the number of vaso-occlusive crises and chronic hemolysis could adversely affect GH-IGF-1-IGFBP-3 axis in SCD patients.

In our population 11.5% of patients had pathological HOMA index. In literature there is evidence of insulin resistance among patients with SCD [10]. A multicenter study by *Fung* et al. [44] revealed that for every 10 years of transfusion therapy, subjects with SCD have a 2.5 times greater probability to develop diabetes (while patients with thalassemia have a double risk). However, there are cases of insulin resistance in patients with normal oral glucose tolerance test. In these patients BMI values were above 85° percentile and none of the patients with normal weight had an insulin resistance condition [22]. However, it is important to point out that our study included a pediatric population with a normal BMI and this could also explain the reduced rate of diabetes. There is currently no agreement on the causes of this complication and further investigations are needed.

We found a case of hypergonadotropic hypogonadism and one case of ovarian failure in HbSS genotype group. The etiology of hypogonadism in SCD is not fully understood yet: in some cases, primitive gonadal failure is related to structural anomalies, resulting from chronic tissue damage associated with chronic anemia condition and local vaso-occlusive crises [45]. According to this hypothesis, our study demonstrated a direct correlation between Hb levels and testosterone average values in males, demonstrating how clinical control can affect reproductive function.

This study has some important limitations. This is a single center study with a small sample size. Secondly there are some confounders factors (i.e. genotype, gender…). The main outcome of this study was to report clinical features of our patients, in a cross sectional way, to better understand the actual prevalence of both metabolic alterations and endocrine complications. Surely, a longitudinal study design of these parameters will provide us more information on the natural history of these complications in SCD. However, it must be considered that in our country SCD is a rare disease and, at the best of our knowledge, this is the first Italian study on these topics. In literature there are very little data available on these condition in pediatric patients with SCD mainly in European Countries. We think that it is important to evaluate these conditions in different environmental setting.

## Conclusion

Subjects with SCD show a high prevalence of metabolic alterations and endocrine complications. However, our results suggest that through the achievement of a good clinical control the SCD patients can obtain a positive impact on growth, metabolic and endocrine function.

Consequently, it is crucial to perform periodic anthropometric and endocrine evaluations, especially during puberty, and to have a comprehensive approach to this disease in order to reduce its long-term complications.

## Additional file

Additional file 1: **Table S1.** Anthropometric parameters in males and females. (DOCX 30 kb)

## Abbreviations

ACS: Acute Chest Syndrome
AIFA: Italian Drugs Agency
AMH: Anti-mullerian hormone
BMI: Body Mass Index
FSH: Follicle-stimulating hormone
FT4: Free thyroxine 4
GH: Growth Hormone
Hb: Hemoglobin
HDL: HDL-cholesterol
HOMA: Homeostasis model assessment
HU: Hydroxyurea
IGF-1: Insulin-like Growth Factor-I
IGFBP-3: Insulin-like Growth Factor Binding Protein 3
IR: Insulin resistance
LDH: Lactate dehydrogenase
LH: Luteinizing hormone
SCD: Sickle Cell Disease
SDS: Standard Deviation Score
TH: Target Height
TSH: Thyroid stimulating hormone
VOC: Vaso-occlusive crises
WHO: World Health Organization
