# Supplementary material for: Endocrine and metabolic complications in children and adolescents with Sickle Cell Disease: an Italian cohort study
Source: BMC Pediatr. 2019 Feb 11;19:56. doi: 10.1186/s12887-019-1423-9 (PMC6371531; doi:10.1186/s12887-019-1423-9)
Supplement: Supplementary file 1 — Table S1. Anthropometric parameters in males and females. (DOCX 30 kb) [file 12887_2019_1423_MOESM1_ESM.docx]

**Table S1.** Anthropometric parameters in males and females

| **ANTHROPOMETRIC PARAMETERS** | **Male** | **Female** | ***P-value*** |
| --- | --- | --- | --- |
|  | **Group (n = 29)** | **Group (n = 23)** |  |
| **Pubertal** | 37.9% | 43.5% | *-* |
| **HbSS genotype %** | 75.5% | 69.5% | *-* |
| **Age (years)** | 11.2±4.6 | 11.1±4.8 | 0.6904 |
| **Height-SDS** | -0.2 ± 1,2 | 0.1 ± 0.9 | 0.5883 |
| **Height-SDS adjusted for TH** | 0.4 ± 1.1 | 0.4 ± 0.6 | 0.3184 |
| **BMI-SDS** | -0.4 ± 1,5 | -0.3 ± 1,5 | 0.1770 |
| **Growth velocity**  **-SDS** | -2.1 ± 2.3 | -0.7 ± 2.5 | 0.1463 |
| **Sitting height/height** | 0.5 ± 0.02 | 0.50 ± 0.02 | 0.8347 |

Data are reported as mean±SD (standard deviation)

Abbreviations: SDS, Standard Deviation; TH, target height; BMI, Body Mass Index.
